# Effects of ∆-9 tetrahydrocannabinol on the small intestine altered by high fructose diet: A Histopathological study

**DOI:** 10.1007/s00418-024-02311-y

**Published:** 2024-08-07

**Authors:** Basak Isildar, Alisa Bahar Beydogan, Ece Koyuturk, Zeynep Mine Coskun Yazici, Meral Koyuturk, Sema Bolkent

**Affiliations:** 1 Department of Histology and Embryology, Cerrahpaşa Faculty of Medicine, Istanbul University-Cerrahpaşa, Istanbul, Turkey; 2 Department of Medical Biology, Cerrahpaşa Faculty of Medicine, Istanbul University-Cerrahpaşa, Istanbul, Turkey; 3 Faculty of Medicine, Otto-Von-Guericke-Universität Magdeburg, Magdeburg, Germany; 4 Department of Molecular Biology and Genetics, Faculty of Arts and Sciences, Demiroglu Bilim University, Istanbul, Turkey

**Keywords:** High fructose diet, ∆-9-Tetrahydrocannabinol, Jejunum, Enterocytes, Electron microscopy

## Abstract

The consumption of fructose is increasing day by day. Understanding the impact of increasing fructose consumption on the small intestine is crucial since the small intestine processes fructose into glucose. ∆9-Tetrahydrocannabinol (THC), a key cannabinoid, interacts with CB1 and CB2 receptors in the gastrointestinal tract, potentially mitigating inflammation. Therefore, this study aimed to investigate the effects of the high-fructose diet (HFD) on the jejunum of rats and the role of THC consumption in reversing these effects. Experiments were conducted on Sprague–Dawley rats, with the experimental groups as follows: control (C), HFD, THC, and HFD + THC. The HFD group received a 10% fructose solution in drinking water for 12 weeks. THC groups were administered 1.5 mg/kg/day of THC intraperitoneally for the last four weeks. Following sacrification, the jejunum was evaluated for mucus secretion capacity. IL-6, JNK, CB2 and PCNA expressions were assessed through immunohistochemical analysis and the ultrastructural alterations via transmission electron microscopy. The results showed that fructose consumption did not cause weight gain but triggered inflammation in the jejunum, disrupted the cell proliferation balance, and increased mucus secretion in rats. Conversely, THC treatment displayed suppressed inflammation and improved cell proliferation balance caused by HFD. Ultrastructural examinations showed that the zonula occludens structures deteriorated in the HFD group, along with desmosome shrinkage. Mitochondria were found to be increased due to THC application following HFD. In conclusion, the findings of this research reveal the therapeutic potential of THC in reversing HFD-related alterations and provide valuable insights for clinical application.

**Graphical abstract:**

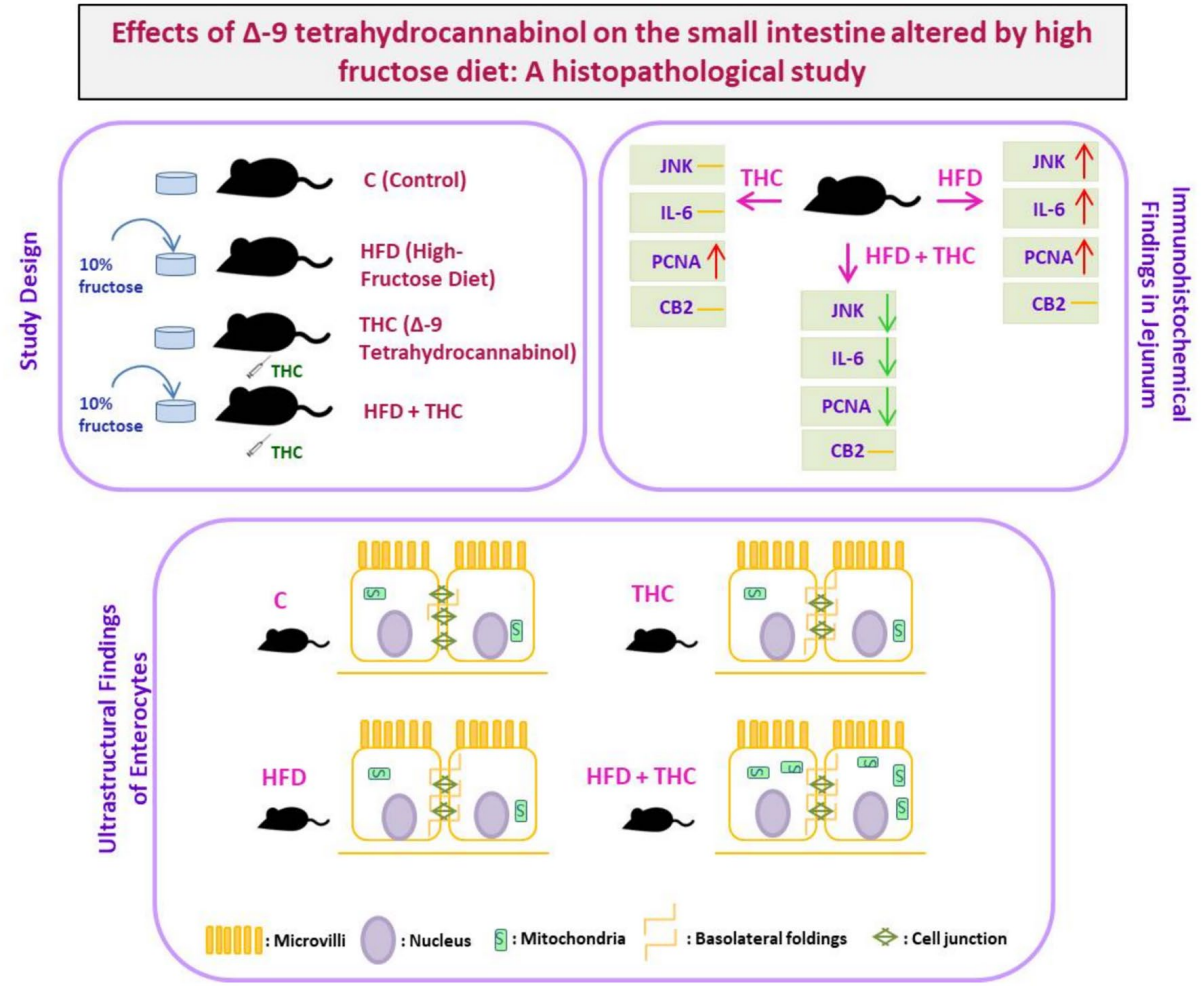

## Introduction

Fructose, a naturally occurring sugar in various fruits, has become increasingly prevalent in modern diets. This surge in fructose consumption can be attributed to the widespread use of sweeteners such as high fructose corn syrup, which are commonly added to sugary beverages and processed food items (Bray 2007; Rizkalla 2010). Through epidemiological studies, a strong association has been revealed between the high fructose diet (HFD) and several diseases, including obesity, non-alcoholic fatty liver disease, type 2 diabetes, and cardiovascular disease (Pan and Kong 2018). Fructose is absorbed into the body through a process facilitated by the fructose transporter, GLUT5. This transport takes place from the brush border of the small intestine to enterocytes. Subsequently, at the basolateral side, GLUT2 enables fructose transport from the enterocytes to the bloodstream (Gouyon et al. 2003). The adverse effects of high fructose consumption, including oxidative stress, inflammation, and autophagy, can lead to tissue and organ dysfunctions (Zhang et al. 2017). So, understanding the connection between high fructose intake and intestinal system dysfunctions is essential, as the intestine is the primary absorption site (Thaiss et al. 2018).

Dysregulation of intestinal homeostasis leads to severe conditions, including inflammatory bowel disease (IBD), celiac disease, and gut-brain interaction diseases such as irritable bowel syndrome. Therefore, maintaining intestinal balance is paramount for overall health. The essential factors in achieving this balance include the intestinal barrier’s functionality and the immune system’s regulation, both of which are regulated through complex signaling mechanisms (Cuddihey et al. 2022). Recent studies indicate that the endocannabinoid system, a signaling system involving lipid mediators and commonly present in the gastrointestinal tract, may serve as a vital connection within the brain-gut-microbiota axis, playing a crucial role in regulating gut homeostasis (Sharkey and Wiley 2016).

Plant-derived cannabinoids and their synthetic analogs could be used medically. ∆-9 tetrahydrocannabinol (THC), a psychotropic constituent, is one of the most investigated plant cannabinoids. THC primarily exerts its effects through cannabinoid (CB) receptors, specifically CB1 and CB2. These receptors are members of the G protein-coupled receptors demonstrated in mammalian tissues and are also represented in the gastrointestinal tract (Pertwee 2001). CB1 receptors are found throughout the gastrointestinal tract, especially in submucosal neurons and myenteric plexus, while CB2 receptors are mainly in inflammatory and epithelial cells (Izzo and Camilleri 2009). Recent evidence suggests CB2 receptors are also present in myenteric and submucosal neurons. Both receptors may have beneficial effects on intestinal inflammation (Gyires and S. Zádori 2016), highlighting the importance of studying THC’s medical use on small intestinal inflammation. The jejunum is crucial for nutrient absorption, and since cannabis affects gastrointestinal motility and secretion, studying the jejunum can reveal how cannabis impacts nutrient uptake and digestive health. With a high concentration of cannabinoid receptors like CB2, which influence gut functions such as motility, inflammation, and pain, the jejunum is ideal for examining cannabis-receptor interactions. Consequently, this study aimed to reveal the inflammatory and structural modifications triggered by HFD in the jejunum. Additionally, considering the anti-inflammatory properties, the possible preventive and therapeutic impacts of THC on the inflammatory and structural changes altered by an HFD in the jejunum were examined by light and transmission electron microscopy.

## Materials and methods

### Experimental design

This study was approved by the Local Ethics Committee on Animal Research of Istanbul University (Project number 2015/66, İstanbul, Turkey). Twenty-four male Sprague–Dawley rats (8–10 weeks old) were housed under a natural light cycle at 22 ± 1ºC. The animals were provided ad libitum access to food and water. One rat was placed in a single cage during the experiment. The animals’ food and fluid consumption was measured daily at the same hour (08.30–09.30 a.m.).

The rats were randomly divided into four groups of six as control (C), high fructose diet (HFD), THC, and HFD + THC. C and THC groups drank tap water. HFD and HFD + THC groups were induced by feeding 10% fructose solution (Merck, 104,007, Darmstadt, Germany) in drinking water for 12 weeks (Sayehmiri et al. 2020). In the last four weeks of the experiment, THC and HFD + THC groups received 1.5 mg/kg/day of THC (THC Pharm, THC-1098, Frankfurt, Germany) with intraperitoneal injection every day for four weeks. Following the treatment period, rats were euthanized under anesthesia induced by a ketamine/xylazine mixture, and jejunal samples were collected for further analysis.

### Histopathological analyses

The jejunum tissues were initially fixed in 10% neutral buffered formalin to prepare them for histological and immunohistochemical examinations. Subsequently, they were dehydrated by passing through a series of increasing alcohol concentrations, embedded in paraffin, and 4 µm sections were taken from the paraffin blocks. These sections were then stained with hematoxylin and periodic acid Schiff (PAS) reaction. The sections underwent an 8-min exposure to periodic acid to reveal aldehyde groups. After washing, the sections were carefully dried and then placed in the dark with the Schiff solution for 15 min. Finally, the nuclei were stained with a 7-min application of hematoxylin and then rinsed in tap water for 10 min. Subsequently, the sections were sequentially passed through increasing concentrations of alcohol, kept in toluene, and closed with an entellan mounting medium. As a result, the nuclei stained purple, and the secretion of goblet cells stained magenta. The sections were examined under an Olympus CX41 light microscope, and mucus secretion activities were analyzed on at least ten villi. All goblet cells were counted, and the percentage of empty cells was calculated (Kunert et al. 2002).

### Immunohistochemical analyses

The jejunum sections were evaluated immunohistochemically for interleukin-6 (IL-6), c-Jun N-terminal kinase (JNK), cannabinoid type 2 (CB2) receptor and proliferating cell nuclear antigen (PCNA) using the streptavidin–biotin-peroxidase technique. Briefly, the sections were deparaffinized in toluene and then rehydrated in the descending ethanol series. The antigen retrieval step was performed with 10 mM citrate buffer (pH 6.0) for 10 × 2 min in the microwave oven (95 °C) and 3% hydrogen peroxide was applied for 10 min to inactivate the endogenous peroxidase activity. After washing, the sections were kept in the blocking solution (Histostain Plus Broad Spectrum Kit, Invitrogen, 859,043, California, USA) for 10 min. Then, the sections were incubated overnight at + 4 °C with the following primary antibodies: IL-6 (sc-1265-R, Santa Cruz Biotechnology, Germany, 1/50 dilution), JNK (sc-7345, Santa Cruz Biotechnology, Germany, 1/20 dilution), CB2 (sc-25494, Santa Cruz Biotechnology, Germany, 1/75 dilution), and PCNA (MS-106-P, Thermo Scientific, USA, 1/50 dilution). Afterward, the sections were washed with phosphate-buffered saline (PBS), kept in the biotinylated secondary antibody solution (Histostain Plus Broad Spectrum Kit, Invitrogen, 859,043, California, USA) for 10 min, and washed again. Streptavidin peroxidase solution (Histostain Plus Broad Spectrum Kit, Invitrogen, 859,043, California, USA) was applied to the sections for 10 min. The detection step was performed with a 3-amino-9-ethyl carbazole (AEC) substrate kit (Invitrogen, 00–2007, California, USA). Next, the sections were counterstained with Mayer’s Hematoxylin. In addition, specificity controls were performed to ensure the specificity of antibodies. Negative controls were included in each staining protocol by replacing the primary antibody with the proper antibody diluent. This allowed us to assess any non-specific binding or background staining. No staining was observed in these negative control sections, confirming the primary antibodies’ specificity. Spleen tissue was used as a positive control for CB2, pancreatic tissue treated with lipopolysaccharide (LPS) was used for IL-6, and pancreatic tissue with pancreatic ductal adenocarcinoma (PDAC) was used for JNK and PCNA.

Each slide was evaluated for ten randomly selected areas regarding the numbers of immunopositive cells and staining intensities. Immunopositive cells in these areas were counted, and the difference between groups was evaluated statistically. The intensity of immunostainings was evaluated semi-quantitatively by the following categories: 0 (negative), + (weakly positive), +  + (positive), and +  +  + (strongly positive). The sections were imaged using a Nikon Eclipse 80i light microscope, and the figures were captured with a Nikon DS-U2 digital camera (Nikon, NY, USA).

### Ultrastructural analyses

Small fragments of the jejunum samples were fixed with 2.5% glutaraldehyde (Merck Millipore, USA) for 1 h, washed with Millonig’s buffer solution, and secondarily fixed with 1% osmium tetroxide (Merck Millipore, 1.00983.2511, USA). Then, samples were dehydrated through the increasing alcohol series and embedded in Araldite (Sigma-Aldrich, Missouri, USA). After polymerization, ultra-thin sections passing through the villous regions were taken and placed on copper grids. These grids were contrasted using uranyl acetate (Merck, 4,087,843, Missouri, USA) and lead citrate (Sigma Aldrich, 15,326-100G, Missouri, USA) (Ozkan et al. 2022). Finally, the analyses were conducted using the Jeol Tem-1011 electron microscope, and micrographs were captured using the Olympus Soft Imaging camera.

### Data analyses

Statistical analyses were conducted using SPSS software (version 21.0, SPSS, IL, USA). Experimental data were presented as the mean ± standard error of the mean (SEM). One-way ANOVA test was performed for multiple comparisons. A value of p < 0.05 was considered statistically significant for all tests. The body weight gain statistical calculations were performed using the GraphPad Prism 5 software. The data were presented as the mean ± SEM for each group and compared with the non-parametric Kruskal–Wallis test.

## Results

### Body weight change results

**Fig. 1:**
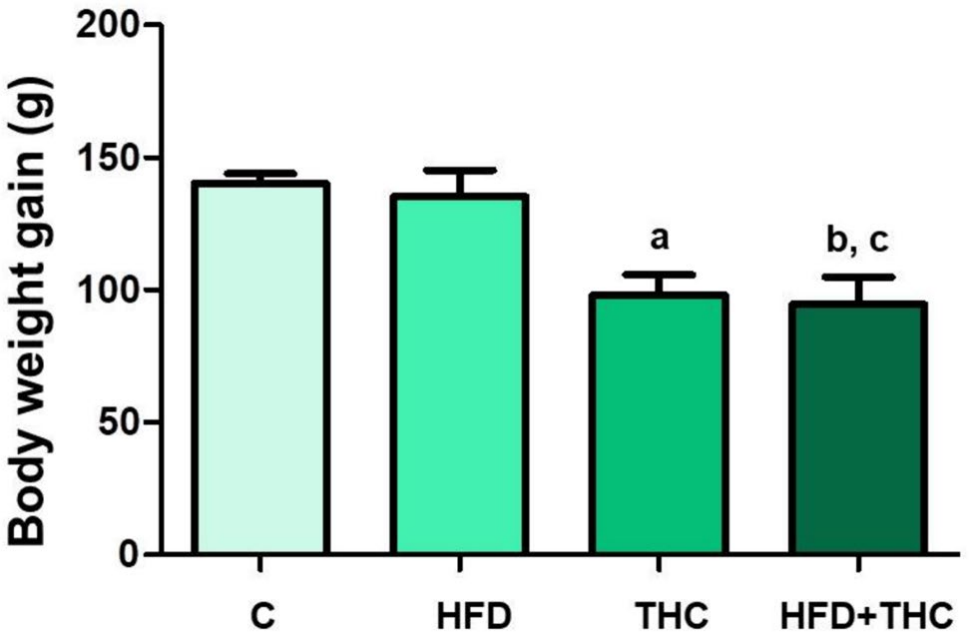
Body weight gains, the beginning and final mean values of body weight were presented. ap < 0.01 vs. the C and the HFD groups, bp < 0.01 vs. the C group, and cp < 0.05 vs. the HFD group

The change in body weight was calculated by subtracting the mean initial value from the mean final value, and there were significant differences between the groups. Accordingly, the THC group exhibited a notable decrease in weight gain compared to both the C (p < 0.01) and the HFD group (p < 0.01). Likewise, the decrease observed in the HFD + THC group was significant compared to both the C (p < 0.01) and the THC group (p < 0.05). The data are presented in Fig. 1.

### Histopathological analyses

**Fig. 2:**
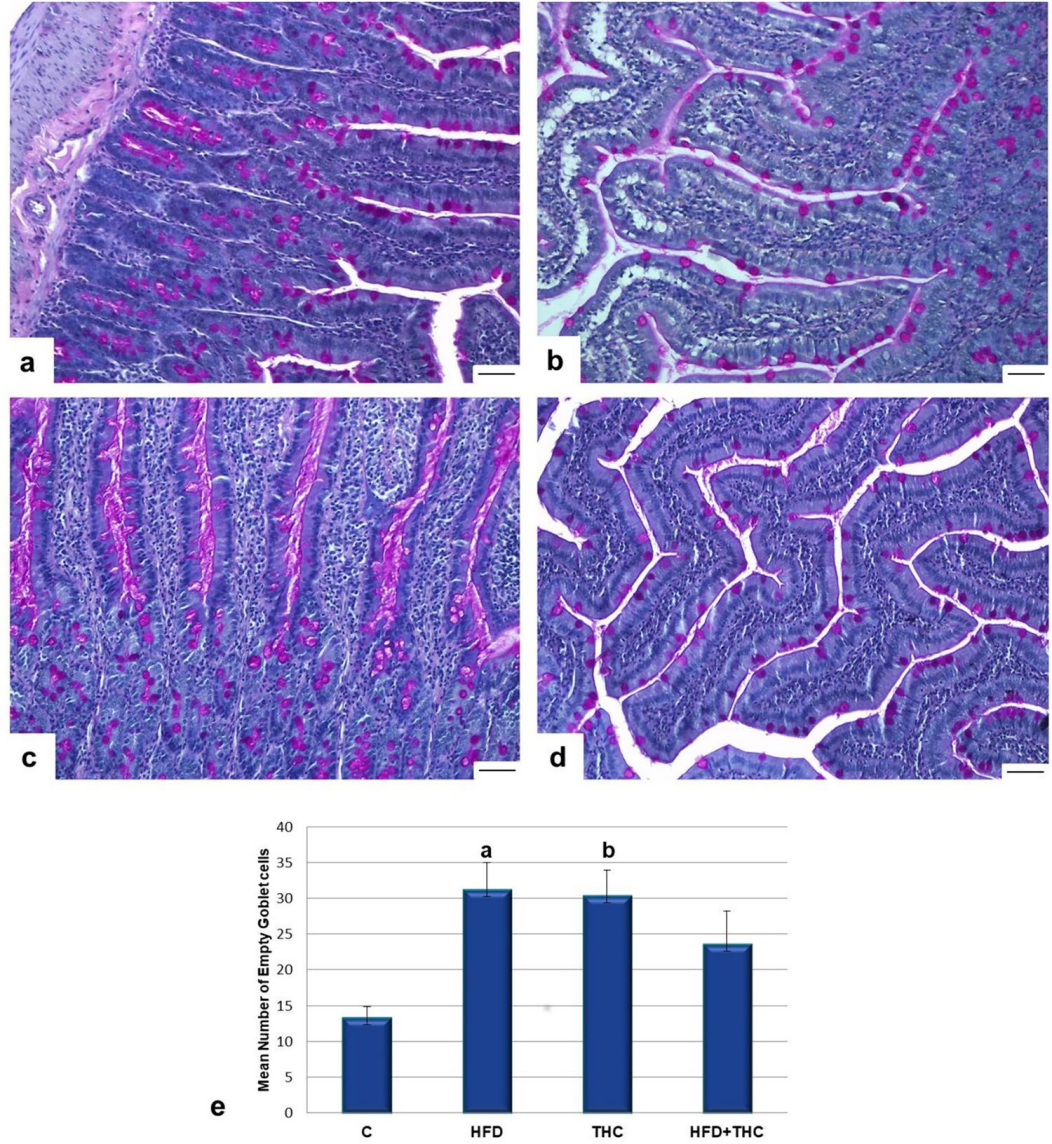
**a**-**d**: The increase in mucus secretion was observed with the PAS reaction in the experimental groups. Histological image of the C group (**a**), HFD group (**b**), THC group (**c**), and HFD + THC group (**d**). PAS + H. **e**: Numbers of empty goblet cells in the groups. a,bp < 0.01 vs. the C group. The bar scale size = 50 µm

No morphological changes were observed in the jejunum’s epithelial integrity or villi structure upon light microscopic examination. Assessment of the ratio of PAS-positive goblet cells for analysis of mucus secretion revealed an increase in the percentage of empty goblet cells on the villi in the experimental groups (Fig. 2a-e). Consequently, a significant increase in mucus secretion was evident in both the HFD and THC groups compared to the C (p < 0.01, p < 0.01). In the HFD + THC group, the rise in mucus secretion was decreased compared to the HFD and THC groups. Statistical analysis showed no significant difference between the C and HFD + THC groups.

### Immunohistochemical analyses

**Fig. 3:**
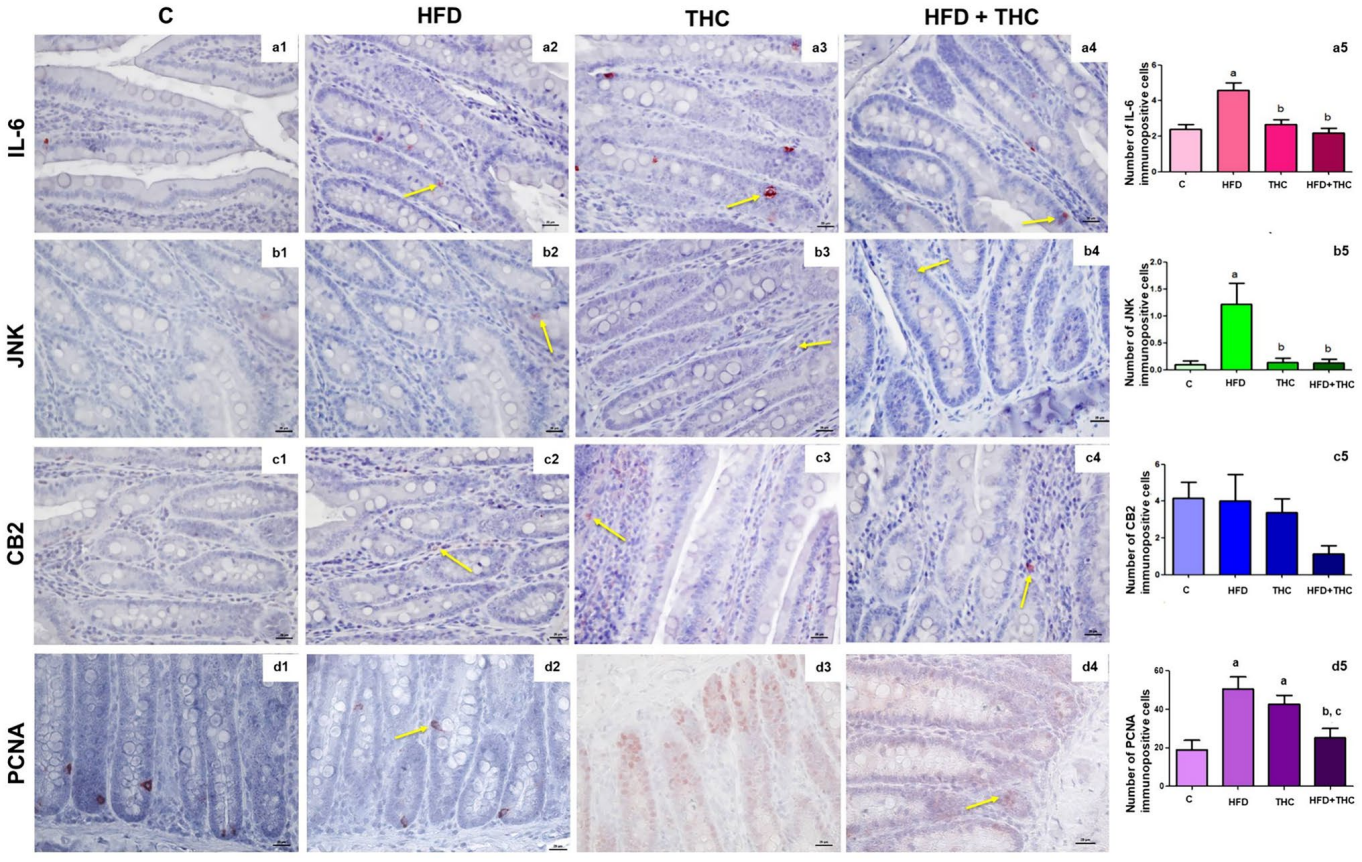
**a1**–**a4**: Representative micrographs for IL-6 immunostaining. **a5:** Numbers of the IL-6 immunopositive cells, ap < 0.001 vs. C group; bp < 0.001 vs. HFD group. **b1**–**b4**: Representative micrographs for JNK immunostaining. **b5:** Numbers of the JNK immunopositive cells, ap < 0.01 vs. C group, bp < 0.01 vs. HFD group. **c1**–**c4**: Representative micrographs for CB2 immunostaining. **c5:** Numbers of the CB2 immunopositive cells, a,b,cp > 0.05. **d1**–**d4**: Representative micrographs for PCNA immunostaining. **d5:** Numbers of the PCNA immunopositive cells, ap < 0.001 vs. C group, bp < 0.001 vs. HFD group, cp < 0.01 vs. THC group. The bar scale size = 20 µm

The immunopositive cell numbers in the jejunum for IL-6, JNK, CB2, and PCNA antibodies and the representative micrographs of the groups are depicted in Fig. 3. The intensity of immunostainings is given in Table 1. Expression levels of IL-6 and PCNA were evaluated in Lieberkühn crypt cells, while JNK and CB2 expressions were localized in the connective tissue of intestinal villi. The HFD group exhibited a significant increase in IL-6 and JNK-immunopositive cells compared to the C group (p < 0.001 and p < 0.01, respectively). Additionally, immunostaining intensities of IL-6 and JNK were higher in the HFD group than in the control group. However, no significant changes were observed in the number and intensity of CB2-immunopositive cells among the groups, except for the HFD + THC group. Finally, significant differences were noted when comparing the PCNA-immunopositive cell rate of the HFD + THC group to the HFD group (p < 0.001) and the THC group (p < 0.01). Notably, immunopositive cells in the HFD + THC group decreased to levels comparable to the control group with THC treatment, with no statistically significant difference observed compared to the control group.

**Table 1:** The intensity of immunostainings

	Control	HFD	THC	HFD + THC
IL-6	** + **	** + + **	** + **	** + **
JNK	** + **	** + + **	** + **	** + **
CB2	** + **	** + **	** + **	** + **
PCNA	** + + + **	** + + + **	** + + + **	** + + + **

### Ultrastructural analyses

**Fig. 4:**
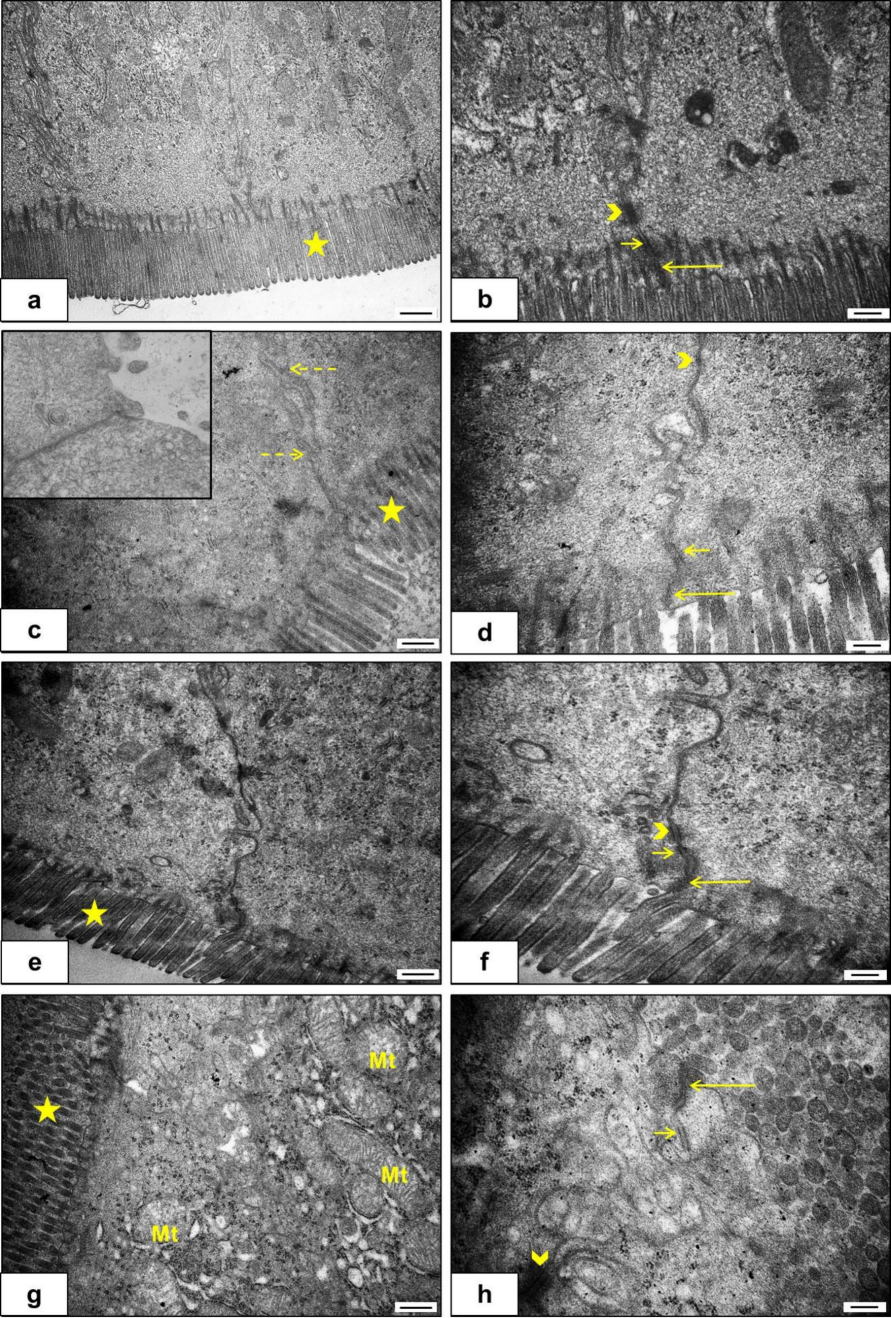
The ultrastructural findings of the groups. **a**–**b**: C group (n = 4), **c**–**d**: HFD group (n = 3), the small square in c: Showing one case which has loss of microvilli on a large number of enterocytes, **e**–**f**: THC group (n = 3), **g**–**h**: HFD + THC group (n = 3). Star: microvilli, Mt: mitochondria, long arrow: zonula occludens, short arrow: zonula adherens, arrowhead: desmosome, dashed arrow: basolateral folds. The bar scale size (a, c, e, g) = 500 nm. The bar scale size (b, d, f, h) = 200 nm

Microvilli morphologies, lateral cellular junctions, mitochondria structure and density, and basolateral folds in the jejunum were evaluated by transmission electron microscopy (Fig. 4). According to the C group, the microvilli length and morphology did not change in the experimental groups, except one case which has loss of microvilli on a large number of enterocytes (the small square in Fig. 4c). Zonula occludens junctions, which establish the intestinal barrier at the apical pole of the lateral membranes of enterocytes, were observed in all groups. However, structural alterations were seen in the HFD group. Electron-dense structures of zonula occludens have deteriorated at several enterocytes in the HFD group, and desmosomal adhesions were examined as shrunken in the HFD and HFD + THC groups than in the C group. The density of mitochondria was elevated in the HFD + THC group. The enterocytes had characteristic membranous basolateral foldings in the C group. According to the C group, these foldings were examined as increased in the THC, HFD, and HFD + THC groups.

## Discussion

This study examined the effects of the HFD on the jejunum in vivo, at the cell and the organelle level, and investigated the role of THC consumption in reversing these effects. Accordingly, at the end of the 12-week experimental period, the examination of weight changes in rats showed no statistically significant change in the HFD group. In contrast, weight gain was reduced in the THC and HFD + THC groups. The direct relationship between HFD and weight gain is still controversial. While some studies indicate that fructose consumption is directly related to weight gain and causes obesity (Pereira et al. 2017; Sohrabi et al. 2018), some studies show that high fructose consumption cannot be associated with weight gain (Castro et al. 2015; Ramos et al. 2017). The lack of weight change despite increased fructose consumption could be explained by the decrease in total calorie intake due to over-fructose consumption. Our study, consistent with these studies, showed that an HFD is not directly related to weight gain. Additionally, the decrease in weight gain in THC and HFD + THC groups draws attention to the regulatory effect of THC on body weight and food intake. Although Brierley et al*.* (Brierley et al. 2017) reported that cannabis purified from the cannabis plant is a novel appetite stimulant, some studies show that regular THC use reduces energy intake in diet-induced obese mice (Cluny et al. 2015). It has been shown that the incidence of obesity in frequent cannabis users is lower than in non-users. In support of this, studies report that lifelong THC use regulates weight gain by showing the same effect as other anorexigenic drugs, such as 3,4-methylenedioxy-N-methylamphetamine (MDMA) (Le Foll et al. 2013). Therefore, it can be thought that chronic THC use prevents body weight gain by reducing total calorie intake.

It has been reported that feeding a high-fructose diet disrupts mucosal integrity (Kumar Jena and Prajapati 2016) and decreases the expression of occludin proteins (Kawabata et al. 2019). The intestinal barrier formed by the zonula occludens has a critical role in the selective permeability of enterocytes and is the main target of research on intestinal pathologies (Suzuki 2020). Preserved and intact desmosomal junctions are suggested as a prerequisite for the integrity of occludens junctions in the intestinal mucosa (Ungewiß et al. 2017). In line with this information, our ultrastructural findings indicated minimal destruction in the intestinal epithelial integrity. Electron microscopic examination revealed that the structure of the zonula occludens had a deteriorated appearance, and the desmosome attachment plates were diminished. However, the damage to the intestinal epithelial integrity may vary depending on the duration and concentration of feeding with the HFD. In addition, the presence of a large number of enterocytes in one case with almost complete disappearance of microvilli indicates that there may be differences in individual responses. It is thought that THC could be used to prevent or reverse the effects of possible ultrastructural epithelial barrier damage caused by an HFD. Goblet cells, the primary source of mucus production, have been studied since the mucus layer is a protective barrier that stretches over the intestinal epithelium (Birchenough et al. 2015). Accordingly, it was observed that mucus secretion increased significantly in the HFD and THC groups. Increased mucus secretion can be considered a protective response to HFD administration. In addition, studies have reported that THC also increases mucus secretion and thus protects the gastric mucosa (Abdel-Salam et al. 2015; Abdel-Salam 2016). On the other hand, the fact that mucus secretion was lower in the HFD + THC group compared to these two groups may be due to the decreased need for protection, as THC began to ameliorate the HFD-induced disruptions on epithelial integrity. Noticeable basolateral membrane folds, another ultrastructural finding, especially in HFD groups, are thought to be related to the transport mechanism of fructose. Because fructose absorption occurs via GLUT5, which is localized in microvilli and then transported via GLUT2 or directly into the basolateral space (Said 2018), this change in the ultrastructure of enterocytes is thought to occur due to increased fructose absorption.

The research suggested that high fructose consumption triggers inflammation in the body (Castro et al. 2015; Crofts 2015). IL-6, which plays an essential role in host defense, is promptly produced by monocytes and macrophages when infections or tissue injuries occur and contributes to the removal of infectious agents and restoration of damaged tissues through activation of immune, hematological, and acute phase responses. When the stress in the host is terminated, IL-6 synthesis stops, but uncontrolled excessive or persistent IL-6 production plays a pathological role in developing various inflammatory diseases and cancers. Therefore, appropriate expression of IL-6 is vital for host defense (Tanaka et al. 2016). In our study, IL-6 was examined immunohistochemically in the jejunum to observe the effects of HFD on inflammation and investigate the role of THC in reversing these effects. According to the findings, there was a significant increase in IL-6 immunoreactivity in the HFD group, indicating that fructose triggers inflammation. It has also been reported that IL-6 production increases mucus secretion by stimulating the mucin genes MUC5B and MUC5AC (Chen et al. 2003). It can be said that the significant increase in mucus secretion in the HFD group may have occurred due to the increased IL-6 level, which caused the triggered inflammation. The significant decrease in IL-6 immunoreactivity in rats treated with THC on HFD compared to the HFD group indicates that the administration of THC may be an effective agent in combating existing inflammation. In addition, PCNA and JNK signaling pathway expressions in jejunum were evaluated immunohistochemically. JNK is one of the central signaling cascades of the MAPK signaling pathway and is involved in apoptotic and non-apoptotic cell death mechanisms in response to harmful extracellular stimuli such as inflammatory cytokines and UV-irradiation (Roy et al. 2008). The findings determined that JNK expression increased significantly in the jejunum sections of the HFD group, and this increase decreased with the THC administration. PCNA is a protein that complexes with cyclin D and cyclin-dependent kinases. It is specifically expressed in proliferating cell nuclei. The intestinal mucosa has a highly renewing cell cycle capacity, and PCNA immunohistochemistry can be used as a valuable marker of the proliferation activity in the intestinal mucosa (Jin et al. 2004; Strzalka and Ziemienowicz 2011; Rees et al. 2020). Diet is reported as an extrinsic factor that affects intestinal stem cell niche during homeostasis. It is suggested that it contributes to the regulation of intestinal regeneration (Santos et al. 2018). However, there are no proven findings on the effects of the high fructose diet on the stem cell niche of the intestinal glands. When our PCNA results were examined, there was an increase in PCNA expression in the HFD and THC groups and a decrease in the HFD + THC group vs. HFD. In this situation, the effect of THC is limited to regulating the enterocyte renewal cycle when impaired with HFD.

Modulation of endocannabinoid system (ECS) activity, which consists of CB1 and CB2 receptors, endocannabinoids, and their synthetic and metabolizing enzymes, may be beneficial in the treatment of many diseases such as inflammatory and cardiovascular diseases, diabetes, obesity, liver diseases, as well as in the amelioration of chemotherapy-related side effects and pain (Gyires and S. Zádori 2016). Therefore, this study evaluated the CB2 receptor, generally found in immune tissues and macrophages, immunohistochemically. The immunopositivity of CB2 receptors decreased in the HFD + THC groups. However, no significant difference was observed between the CB2 expression levels in the groups.

In conclusion, our results show that high fructose consumption causes inflammation in the jejunum, increases mucus production, and disrupts the balance of cell proliferation. It has been determined that THC application is efficient in reversing these effects. In this context, new findings have been presented that THC can be a candidate as a therapeutic agent.

## Data Availability

The datasets used and/or analyzed during the current study are available from the corresponding author on reasonable request.
